# Peripheral immune signature resembles tumor microenvironment and predicts clinical outcomes in head and neck squamous cell carcinoma

**DOI:** 10.3389/fimmu.2022.915207

**Published:** 2022-09-06

**Authors:** Zixuan Hu, Jiaying Zhou, Yupeng Li, Yizhao Luan, Huan Li, Bo Jia, Zhi Xie, Bin Cheng, Tong Wu

**Affiliations:** ^1^ Hospital of Stomatology, Sun Yat-sen University, Guangzhou, China; ^2^ Guangdong Provincial Key Laboratory of Stomatology, Guangzhou, China; ^3^ Guanghua School of Stomatology, Sun Yat-sen University, Guangzhou, China; ^4^ State Key Laboratory of Ophthalmology, Zhongshan Ophthalmic Center, Sun Yat-sen University, Guangzhou, China; ^5^ Department of Intensive Care Unit (ICU), Sun Yat-sen University Cancer Center, State Key Laboratory of Oncology in South China, Collaborative Innovation Center for Cancer Medicine, Guangzhou, China; ^6^ Department of Oral Surgery, Stomatological Hospital, Southern Medical University, Guangzhou, China

**Keywords:** head and neck squamous cell carcinoma (HNSCC), prognostic immune signature, neutrophil-to-lymphocyte ratio (NLR), tumor immune microenvironment (TIME), tumor-infiltrating immune cell

## Abstract

The contour of the tumor immune microenvironment (TIME) is very important for tumor prognostic prediction but hard to be characterized in clinical practice. It is unclear practice whether the peripheral immune signature (pIS) reflects the TIME as a feasible prognostic indicator for head and neck squamous cell carcinoma (HNSCC) patients. Here, we enrolled 599 HNSCC patients from three domestic institutes to explore the relationship between the pIS and survival. The peripheral neutrophil-to-lymphocyte ratio (pNLR) was screened out as a significant prognostic variable through multivariable COX regression analyses. An inverse correlation between pNLR and survival was found in the data of these 599 patients. Meanwhile, the bulk tumor RNA-seq data of 913 cases were downloaded from The Cancer Genome Atlas (TCGA) and Gene Expression Omnibus (GEO) databases to identify the prognosis-associated TIME features. The TIME feature was consistent to the finding of clinical data, in which high tissue NLR predicted a poor prognosis. Differentially expressed immune-related gene (DEIRG) enrichment analysis also showed a trend that the gene sets in patients with a good prognosis were enriched in lymphocyte-related functions, while those with a poor prognosis were enriched in neutrophil-related functions. At the same time, the well prediction performance of our model based on DEIRGs was verified in both TCGA and GEO cohorts. Finally, the correlation between pIS and the TIME was confirmed in a small independent cohort of 30 HNSCC patients. A positive correlation was confirmed prospectively between the pNLR and the TIME pattern in our independent cohort. Our findings provide evidence that the pNLR is a feasible prognostic signature that reflects the TIME patterns to some extent in HNSCC.

## Introduction

Head and neck squamous cell carcinoma (HNSCC) accounts for more than 90% of head and neck cancer cases with 878,000 new cases and 445,000 deaths in 2020 (1). The prediction of HNSCC patients’ survival is essential for counseling, preoperative risk assessment, treatment planning, and post-therapeutic monitoring (2, 3). Many prognostic factors, including tumor histopathological features, peripheral indicators, tumor immune microenvironment (TIME), tumor-related gene expression, and genetic alterations have been utilized to predict HNSCC survival (4–7).

Intuitively, the characteristics of the TIME are intricate networks relevant to tumor clinical outcomes. The distribution of tumor-infiltrating immune cells (TIICs) and secretion of cytokines and chemokines in the TIME are key players affecting tumor growth and progression, as well as patient prognoses (8–10). For instance, the high density of tumor-infiltrating neutrophils reflects an immunosuppressive state and thus results in detrimental outcomes in tumor progression (11–15). In contrast, high CD3(+) and CD8(+) lymphocyte infiltration is suggestive of good survival (7, 11, 16, 17). Derived non-cellular components in the TIME such as interleukin-1β (IL-1β), interleukin-6 (IL-6), and interleukin-8 (IL-8) are also found to be strongly associated with poor clinical outcomes, while interferon-γ (IFNγ) refers to good outcomes (18). These clinical associations make the characterization of the TIME hold a great promise as prognostic biomarkers for solid tumors in clinical management (11, 19). However, it is difficult and time-consuming to picture the complex TIME in HNSCC patients. A feasible convenient approach to predict the TIME and tumor prognosis in HNSCC is urgently needed.

The peripheral immune signature (pIS) is frequently used to measure a systemic immune state of patients with tumors (5, 20, 21) and of great clinical value in predicting the prognosis of solid tumors (5, 22, 23). For example, a high peripheral neutrophil-to-lymphocyte ratio (pNLR) was proven to be associated with the adverse survival of patients in various solid tumors, including melanoma, colorectal cancer, and gastric cancer (5, 23–26). Meta-analysis found that an elevated preoperative pNLR was prone to a poor prognosis and local invasion in head and neck cancer (27). The pNLR could be extensively used in clinical practice because of the easy accessibility and low cost of the preoperative routine blood cell count required by the pIS method (28). Nevertheless, the underlying reasonability and biological relevance regarding its prognosis value are largely unknown.

In view of these findings that both the pIS and TIICs were associated with tumor prognoses, it is undetermined whether and to what extent they are associated with each other in HNSCC. Some studies have investigated the prognostic impact of the pNLR or NLR-related TIME, respectively, but they did not combine the two indicators to discover their interconnections (29, 30). In this study, the multicenter clinical data of HNSCC patients were gathered to find the promising peripheral prognostic signature and estimate its prognostic value. Public databases were collected to explore the relationship between the TIME and HNSCC patients’ survival in several aspects, including TIICs, immune-related function, and differentially expressed genes (DEGs). Further, paired pIS and tumor samples were analyzed to confirm the correlation between the pIS and TIICs and their prognostic value. We hope to provide evidence for the potential mechanisms underlying the predictive value of pIS for HNSCC patients.

## Materials and methods

### Patients and clinical data collection

The clinical records of 3,985 HNSCC patients from three domestic institutes between 2000 and 2018 were retrospectively reviewed. An eligible inclusion meeting the criteria as follows: (I) available preoperative data including clinical data (demographic and tumor characteristics), pathological data [pathological tumor and lymph node (TN) stage and tumor differentiation grade], and blood routine examination data; (II) received curative-intent surgical resection; and (III) postoperative pathological confirmation as HNSCC. Patients were excluded according to the following criteria: (I) experienced distant metastasis at the time of diagnosis and (II) with a previous history of HNSCC or other malignancy treatment.

Five hundred ninety-nine patients were included in total, in which 138 were from the Hospital of Stomatology, Sun Yat-sen University (SYSU), 402 were from the SYSU Cancer Center, and 59 were from the Guangdong Provincial Stomatology Hospital. The study was approved by the Ethics Committee of Hospital of Stomatology, SYSU.

Clinical data included demographic (gender, age, smoking history) and tumor characteristics (tumor site). Pathological data included the pathological tumor and lymph node (TN) stage and pathological tumor differentiation grade. Blood parameters were collected from preoperative routine blood tests, including pNLR and the peripheral lymphocyte-to-monocyte ratio (pLMR). The tumor pathological TN stage was classified according to the 8th edition of the American Joint Committee on Cancer (AJCC) staging system for HNSCC (31). Overall survival (OS) was regarded as the endpoint, determined by the date from surgery to the last follow-up or death.

### Cox regression–based feature selection and prognostic analyses

Univariable and multivariable Cox regression was performed to screen the independent prognostic factors affecting OS. The qualified prognostic factors with significant differences in the univariable analysis were incorporated into the multivariable analysis. Full and reduced models were constructed to reconfirm the significant predictive value of the selected independent prognostic factors. The time-dependent receiver operating characteristic (ROC) curve and Kaplan–Meier survival curves were used to evaluate the independent prognostic factors (picked out by multivariable Cox regression) and the models, using Wilcoxon tests, with timeROC and survminer packages in R. Two different-level groups were divided into high- or low- groups with the cutoff value by the R package ‘maxstat’. The C-index was calculated and compared with the survcomp package to evaluate the prognostic performance of the models.

### Public RNA-sequencing data collection, processing, and analyses

The gene expression profiles of frozen tumor tissue samples and clinical records from public head and neck cancer cohorts were retrospectively analyzed. The training cohort was downloaded from The Cancer Genome Atlas (TCGA; https://portal.gdc.cancer.gov/) with 546 head and neck cancer samples (502 tumor and 44 normal), and the validation cohort was taken from the National Center for Biotechnology Information (NCBI) Gene Expression Omnibus (NCBI-GEO; http://www.ncbi.nih.gov/geo/; GSE41613 with 97 head and neck cancer samples and GSE65858 with 270 samples). The expression profile of the RNA-seq data from the TCGA was transformed to reads per kilobase million reads (RPKM), and the normalized results of the microarray were used directly.

We defined patients who survived longer than 3 years after surgery as good-prognosis patients and those who survived no longer than 3 years after surgery as poor-prognosis patients in the TCGA cohort. The survival population with a follow-up duration less than 3 years was not included in grouping.

### Tumor immune cell–infiltrating characteristics

RNA-seq data from the TCGA data set was transformed to the proportion of 22 types of TIICs by the CIBERSORT deconvolution algorithm (32). The 22 cell types included B cells, T cells, natural killer cells, macrophages, dendritic cells, eosinophils, and neutrophils, among others. The formula of the tumor infiltrating neutrophil-to-lymphocyte ratio (tNLR) is neutrophil / (T cell + B cell + NK cell).

### Differentially expressed immune-related gene collection and functional enrichment analysis

Genes were screened to have sufficient expression (mean RPKM > 1 and detected in more than 60% samples). The differentially expressed genes (DEGs) between tumors with good and poor prognoses were identified with DESeq2 (*p* < 0.05 and |log2(fold change) | > 0.8) (33). Immune-related genes (IRGs) were obtained from the Immport database (https://www.immport.org/shared/genelists) and shown as **Table S4**. The IRGs downloaded were filtered with the TCGA gene expression list. The DEIRGs were filtered with DEGs and IRGs.

Gene Ontology (GO) term and Kyoto Encyclopedia of Genes and Genomes (KEGG) pathway enrichment analyses were performed with the clusterProfiler package to explore the different immune states (*p* < 0.05) (34). GO enrichment included the biological process (GO_BP), cellular component (GO_GC), and molecular function (GO_MF).

### Constructing risk assessment gene model and nomogram

The DEIRGs were filtered with univariable Cox regression and then ranked by the random forest algorithm using the randomForestSRC R package to further narrow the genes, and an immune gene–based risk score was constructed using the selected important genes. Thereinto, the risk score was calculated using the expression of DEIRGs, which were transformed to log2(RPKM) and then scaled to 1 million counts in each sample to make the model we constructed usable for different datasets or platforms and weighted by the corresponding coefficients derived from multivariable Cox regression. The model discriminative ability was evaluated by ROC and Kaplan–Meier survival analysis with timeROC and survminer packages in R. A nomogram was constructed based on the model for clinical application with the rms package in R. The total risk points of each patient were calculated according to the established nomogram as final predictors and calibration plots were used to verify the nomogram.

### Immunohistochemical staining validation

The formalin-fixed paraffin-embedded (FFPE) tumor specimens from 30 patients with a paired pIS were enrolled for immunohistochemical (IHC) staining. CD3 was used as a marker for T cells, CD11b for myeloid cells, and CD19 for B cells, respectively. Briefly, paraffin sections were deparaffinized in xylene, rehydrated, and exposed to 3% hydrogen peroxide. The CD3 antibody (Abcam Cat# ab16669, RRID : AB_443425), CD11b antibody (Abcam Cat# ab133357, RRID : AB_2650514), and CD19 antibody (Abcam Cat# ab134114, RRID : AB_2801636), incubated with tumor sections in a humidified chamber at 4°C overnight, followed by the secondary antibody (Gene Tech Cat# GK5007, RRID : AB_2904222) for an hour. Slides were counterstained with hematoxylin and finally mounted in a non-aqueous mounting medium. Negative controls were included in each staining run.

IHC results were independently assessed by two oral pathologists who were blinded to the relevant clinical data. Three representative fields in each section were selected at ×400 magnification after the initial screen under a microscope at low-power field (×100). The number of total cells and positive-stained cells were identified using ImageJ and averaged for each slide.

### Statistical analyses

Descriptive statistics were used to summarize patients’ clinical characteristics. The Mann–Whitney U test was used to compare the cell type compositions of tumor-associated immune cells between good- and poor-prognosis groups and IHC results. All analyses were conducted using Python and R. All statistical tests were two sided with statistical significance defined with *p* < 0.05.

## Results

### Peripheral neutrophil-to-lymphocyte ratio can predict clinical outcomes of head and neck squamous cell carcinoma patients

**Figure 1:**
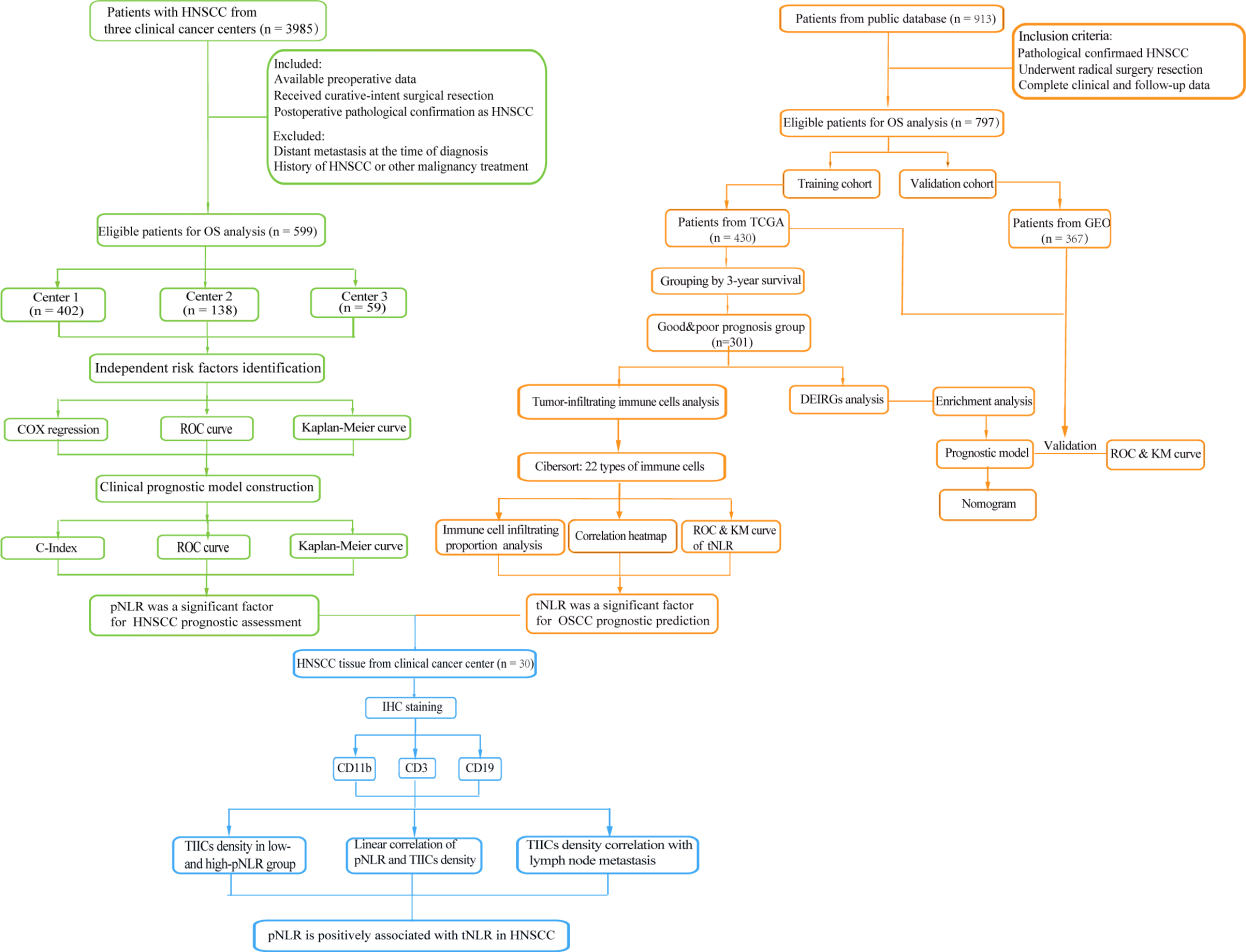
Flowchart of this study. A total of 599 patients from three clinical centers and 913 patients from a public database were collected to respectively identify the prognostic peripheral immune signature (pIS) and prognosis-associated tumor immune microenvironment (TIME) feature. The correlation between the pIS and the TIME was confirmed in a 30 head and neck squamous cell carcinoma (HNSCC) patients’ cohort.

With a unified and strict inclusion strategy (see *Methods*; **Figure 1**), we enrolled a total of 599 HNSCC patients (395 men and 204 women) from three cancer centers. Four hundred forty-six of these 599 patients were alive, and 153 were dead, subjected to a regular follow-up for 49.8 months in average. The summarized characteristics of the 599 HNSCC patients are described in **Table S1**.

We firstly performed univariable Cox regression analyses to screen the potential variables associated with HNSCC survival and found that the age, pathology TN stage, tumor pathology differentiation grade, pNLR, and pLMR individually showed significant association with the OS (*p* < 0.05; **Table 1**). Moreover, we found correlation to varying degrees between these variables, similar with previous studies showing that some of these variables were clinically relevant (35, 36). Using a multivariable analysis, we thus screened the independent variables by adjusting the redundancy raised by multicollinearity. Age (HR: 1.015; 95%CI: 1.002–1.028; *p* = 0.025), pathology N-stage (HR: 2.221; 95% CI: 1.4–3.523; *p* < 0.01), and pNLR (HR: 1.096; 95% CI: 1.011–1.189; *p* = 0.025) were found to be independent predictive factors for OS (**Table 1**).

**Table 1:** Univariable and multivariable Cox regression of the correlation factors of survival.

Factor	Univariable analysis			Multivariable analysis		
	HR	CI (95%)	*p*_value	HR	CI (95%)	*p*_value
Gender	0.710	0.5-1.008	0.055			
Age (year)	1.015	1.003-1.028	0.018	1.015	1.002–1.028	0.025
Smoking history	1.101	0.727-1.666	0.650			
T stage	3.933	2.839-5.449	0.000			
N stage	4.422	3.194-6.122	0.000	2.221	1.4–3.523	0.001
Tumor pathology stage	4.362	3.16-6.02	0.000			
Tumor pathology grade	2.466	1.333-4.563	0.004			
pNLR	1.247	1.17-1.329	0.000	1.096	1.011–1.189	0.025
pLMR	0.781	0.703-0.866	0.000			

**Figure 2:**
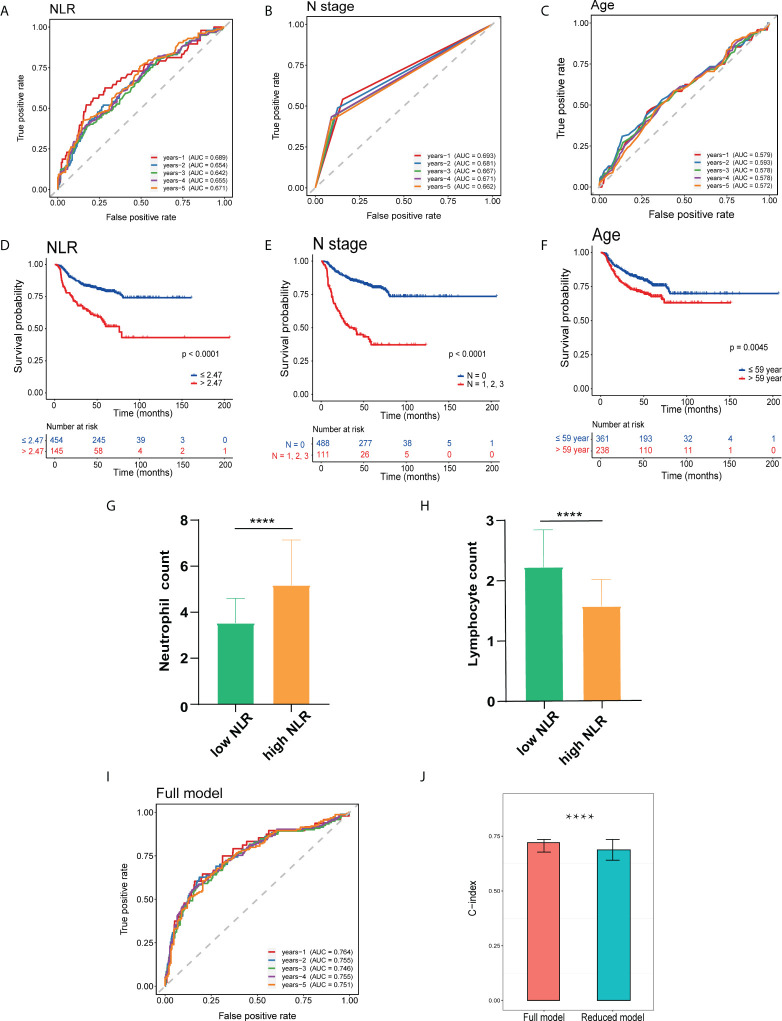
Prognostic analysis of HNSCC independent risk factors. **(A–C)** Receiver operating characteristic (ROC) curve of patients’ preoperative peripheral neutrophil-to-lymphocyte ratio (pNLR) **(A)**, pathology N stage **(B)**, and age **(C)** on OS at 1–5-year follow-up. **(D–F)** Kaplan–Meier overall survival (OS) curves of patients’ preoperative pNLR **(D)**, pathology N stage **(E)**, and age **(F)**. **(G, H)** Peripheral neutrophil **(G)** and lymphocyte **(H)** count in high- and low-pNLR groups. **(I)** ROC curves of the full model (including pNLR, age, and pathology N stage) on OS at 1–5-year follow-up. **(J)** C-index of full and reduced models. ****p ≤ 0.0001.

The area under the curve (AUC) of 1–5-year ROC curves for OS prediction using the pNLR were 0.689, 0.654, 0.642, 0.655, and 0.671, respectively, of which the accuracy was close to those using the pathology N stage (0.693, 0.681, 0.667, 0.671, and 0.662, respectively) and superior to those by the age (0.579, 0.593, 0.578, 0.578, and 0.572, respectively), indicating good sensitivity and specificity for the pNLR (**Figures 2A–C**). As observed in the Kaplan–Meier survival curves (**Figures 2D–F**), patients with a low-pNLR of no more than 2.47 (cutoff value) had better survival, whereas patients with a high-pNLR of more than 2.47 survived shorter (log rank test, *p* < 0.0001). Patients without lymph node metastasis had better survival than those with lymph node metastasis (log rank test, *p* < 0.0001). The older patients (age > 59 years old) had poorer survival comparing with the younger ones (age ≤ 59 years old) (log rank test, *p* = 0.0045). The neutrophil count of the high-pNLR group was significantly higher while the lymphocyte count was lower than that of low-pNLR group (Mann– Whitney U test, *p* < 0.0001), which suggested that the pNLR change was caused both by the neutrophil and lymphocyte counts (**Figures 2G, H**). The distribution of pNLR is shown in **Figure S1**.

A full model including three factors: pNLR pathology N stage, and age, and a reduced model containing two factors: age and pathology N stage, were constructed. Time-dependent ROC on OS at a 1–5-year follow-up C-index was respectively performed. The 1–5-year AUC values of the full model for OS prediction were 0.764, 0.755, 0.746, 0.755, and 0.751, respectively, which were superior to the reduced model (0.721, 0.722, 0.710, 0.716, and 0.706, respectively) (**Figure 2I**, **Figure S2**). In addition, the C-index for the full model (0.721) was significantly superior to the reduced model (0.687), suggesting that the pNLR was a significant prognostic factor (**Figure 2J**).

Collectively, the pNLR was a significant factor for HNSCC prognosis prediction, with the elevated pNLR associated with poor survival.

### Tumor-infiltrating neutrophil-to-lymphocyte ratio in head and neck squamous cell carcinoma associated with tumor prognosis

We next investigated the TIME in HNSCC tumors. A total of 430 HNSCC patients from TCGA data sets (training cohort), 97 patients from GSE41613, and 270 patients from GSE65858 (validation cohort collected from GEO) were enrolled (**Figure 1**; see *Methods*). Patients in the TCGA cohort were divided into good- and poor-prognosis groups according to the 3-year cutoff value (n=301; see *Methods*).

**Figure 3:**
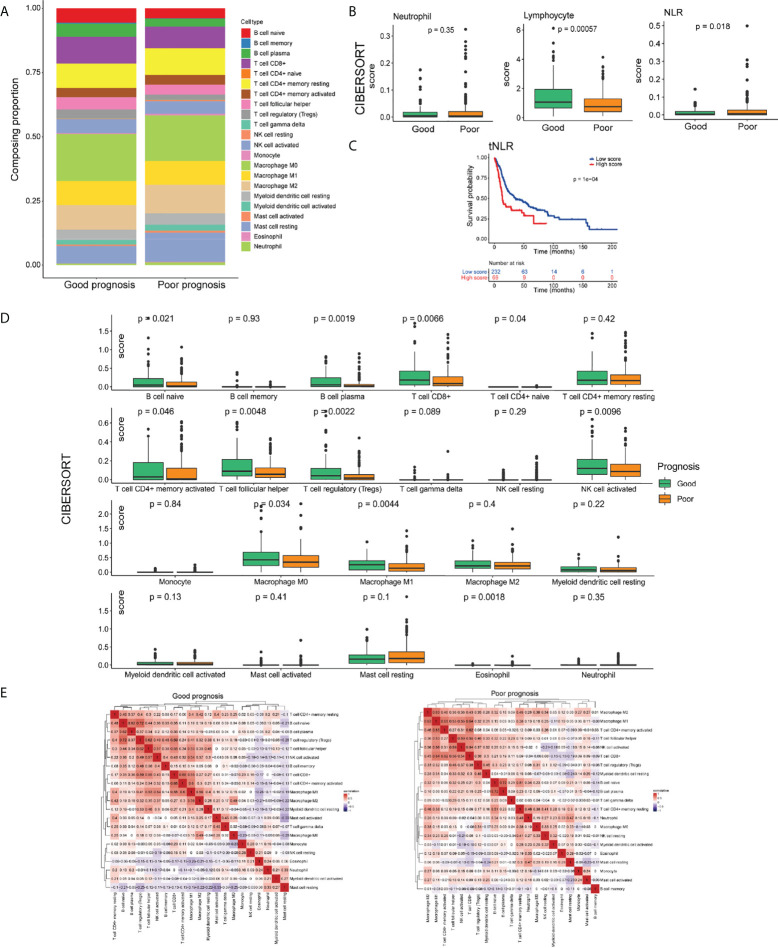
The landscape of the TIME in the HNSCC of good- and poor-prognosis groups. **(A)** Bar charts presenting the proportions of tumor-infiltrating immune cells (TIICs) in good- and poor-prognosis groups. **(B)** Box plots demonstrating the proportion of neutrophils, lymphocyte infiltration, and the tNLR in good- and poor-prognosis groups. **(C)** Box plots of 22 types of TIICs in good- and poor-prognosis groups. **(D)** Correlation heat maps demonstrating the correlation of 22 types of TIICs in good- and poor- prognosis groups. **(E)** Kaplan–Meier OS curves of patients with low and high tNLR.

In total, we identified 22 immune cell subtypes, which showed a statistically global difference between tumors with good and poor prognoses (**Figure 3**). Consistent with the pNLR results, the tNLR also showed significant differences between two prognosis groups. In the good-prognosis group, the tumor-infiltrating lymphocyte proportion was higher than the poor ones, whereas the tNLR was lower than the poor ones (Mann– Whitney U test, *p* = 0.018; **Figure 3B**). The Kaplan–Meier curve showed that patients with a low tNLR proportion had better survival than patients with a high tNLR proportion (**Figure 3** **C** **)**. In addition, B-cell naive, B-cell plasma, CD4(+) memory T cell, CD8(+) T cell, T-cell follicular helper, Tregs, NK-cell activated, and macrophage M0 and M1 were found significantly more abundant in the good-prognosis group than in the poor- prognosis group (Mann–Whitney U test, p < 0.05) (**Figure 3D**).

The positive or negative correlation of immune cell expression was greatly different in good- and poor-prognosis groups. In the good-prognosis group, B-cell naïve and Treg proportion had a strong positive correlation (Pearson correlation, r = 0.72, *p* < 0.05). Meanwhile, in the poor-prognosis group, macrophages had a strong positive correlation with CD4+ and CD8+ T cells (Pearson correlation, r = 0.65, 0.64, respectively, *p* < 0.05) (**Figure 3E**).

To sum up, there were significant differences on the tumor immune pattern between tumors with good and poor prognoses. Higher lymphocytes and a lower NLR-infiltrating proportion referred to a better survival in HNSCC.

### Identification of differentially expressed immune-related genes

**Figure 4:**
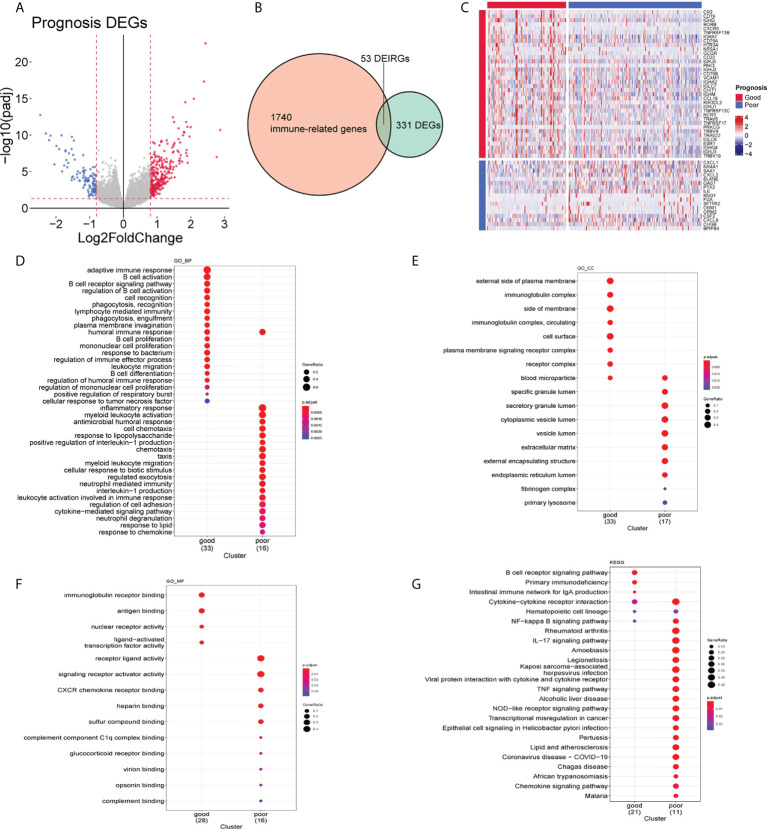
Molecular characteristics of good- and poor-prognosis subgroups. **(A)** Differentially expressed genes (DEGs) of good- and poor-prognosis groups in the The Cancer Genome Atlas (TCGA) data set. **(B)** DEIRGs identified by the intersection of DEGs and IRGs. **(C)** Expression levels of 53 DEIRGs of HNSCC tissues in good- and poor-prognosis groups. **(D–F)** Gene Ontology (GO) enrichment plots of good- and poor-prognosis groups on the biological process **(D)**, cellular component **(E)**, and molecular function **(F)**. **(G)** Kyoto Encyclopedia of Genes and Genomes (KEGG)-pathway enrichment plot of good- and poor-prognosis groups.

By comparing gene expression in tumors with a good prognosis to that in tumors with a poor prognosis, we identified a total of 384 DEGs, of which 276 were upregulated and 108 were downregulated (**Figure 4A**). In view of the different TIME between these two groups (**Figure 3**), we hypothesized that immune-related genes were involved in this systemic microenvironmental change. We collected 384 DEGs and 1,793 IRGs (see *Methods*), among which 53 were DEIRGs (**Figure 4B**). These DEIRGs also showed distinct expression patterns in patients with good and poor prognoses. Notably, we found that lymphocyte function–related genes, such as *CR2* and *CD19*, were increased at the mRNA level in the tumors with a good prognosis, while the mRNA levels of myeloid cell function–related genes, such as *CXCL1* and *IL6*, were increased in the tumors with a poor prognosis (**Figure 4C**). Functional enrichment analyses also showed that the DEIRGs identified in the good-prognosis group were enriched in the terms and pathways related to B-cell activation and the lymphocyte-mediated immunity process, while the DEIRGs identified in the poor-prognosis group were enriched in terms and pathways related to myeloid leukocyte activation and chemotaxis (**Figures 4D–G**). These results collectively suggested an involvement of both lymphocytes and neutrophils in the changed TIME in HNSCC.

### Prognostic model establishment and nomogram construction

**Figure 5:**
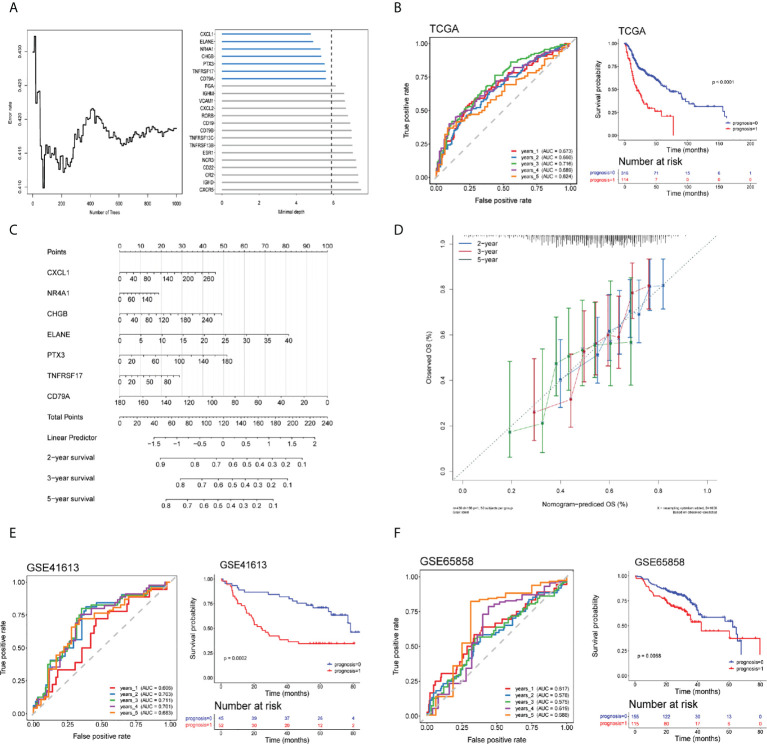
Establishment of a risk assessment model using differentially expressed immune-related genes (DEIRGs). **(A)** Random forest algorithm of DEIRGs for further model gene selection. **(B)** ROC and Kaplan–Meier curves of seven DEIRGs-based model on OS at 1–5-year follow-up in the TCGA training cohort. **(C, D)** Nomogram based on the seven DEIRGs-based model **(C)** and calibration curve for the prediction 2-, 3-, and 5-year OS **(D)**. **(E, F)** ROC and Kaplan–Meier curves of the seven DEIRGs-based model on OS at 1–5-year follow-up in the Gene Expression Omnibus (GEO) validation cohort-GSE41613 **(E)** and GSE65858 **(F)**.

The DEIRGs were further subjected to random forest analysis for selecting the important genes associated with the changed TIME in HNSCC (**Figure 5A**). Seven DEIRGs were finally screened out with the most conservative threshold 5.8902 calculated by randomForestSRC. The prognostic model was constructed based on the seven genes with six high-risk genes and one protective gene. The risk score was defined following the formula: expression level of (0.0038) *CXCL1*+ (0.0037) *CHGB*+ (0.0029) *NR4A1*+ (0.0387) *ELANE*+ (0.0037) *PTX3*+ (-0.0082) *CD79A*+ (0.0022) *TNFRSF17*. For model validation, we added the data of the survival population with a follow-up duration less than 3 years into the TCGA cohort. The 1–5-year AUC values for OS prediction were 0.673, 0.660, 0.716, 0.689, and 0.624, respectively and there was a significant difference between high- and low- risk groups (log rank test, *p* < 0.0001) (**Figure 5B**).

A nomogram based on the seven DEIRG-based model on 2-, 3-, and 5-year OS was constructed, of which a higher score was associated with a poor prognosis (**Figure 5C**). COX regression in the TCGA cohort confirmed the model significantly associated with OS (**Table S2**). Calibration curves for the prediction 2-, 3-, and 5-year OS demonstrated a good agreement between the prediction and observation values (**Figure 5D**).

The GEO validation cohort of the seven DEIRG-based model also performed well in the ROC curve (GSE41613-1-to-5 year AUC values were 0.606, 0.703, 0.711, 0.701, and 0.683, respectively; GSE65858-1-to-5-year AUC values were 0.617, 0.578, 0.575, 0.619, and 0.688, respectively) and was of significant difference between high- and low-risk groups (log rank test, GSE41613, *p* = 0.0002; GSE65858, *p* = 0.0068) (**Figures 5E, F**).

### Correlation between peripheral and tumor-infiltrating immune cells

**Figure 6:**
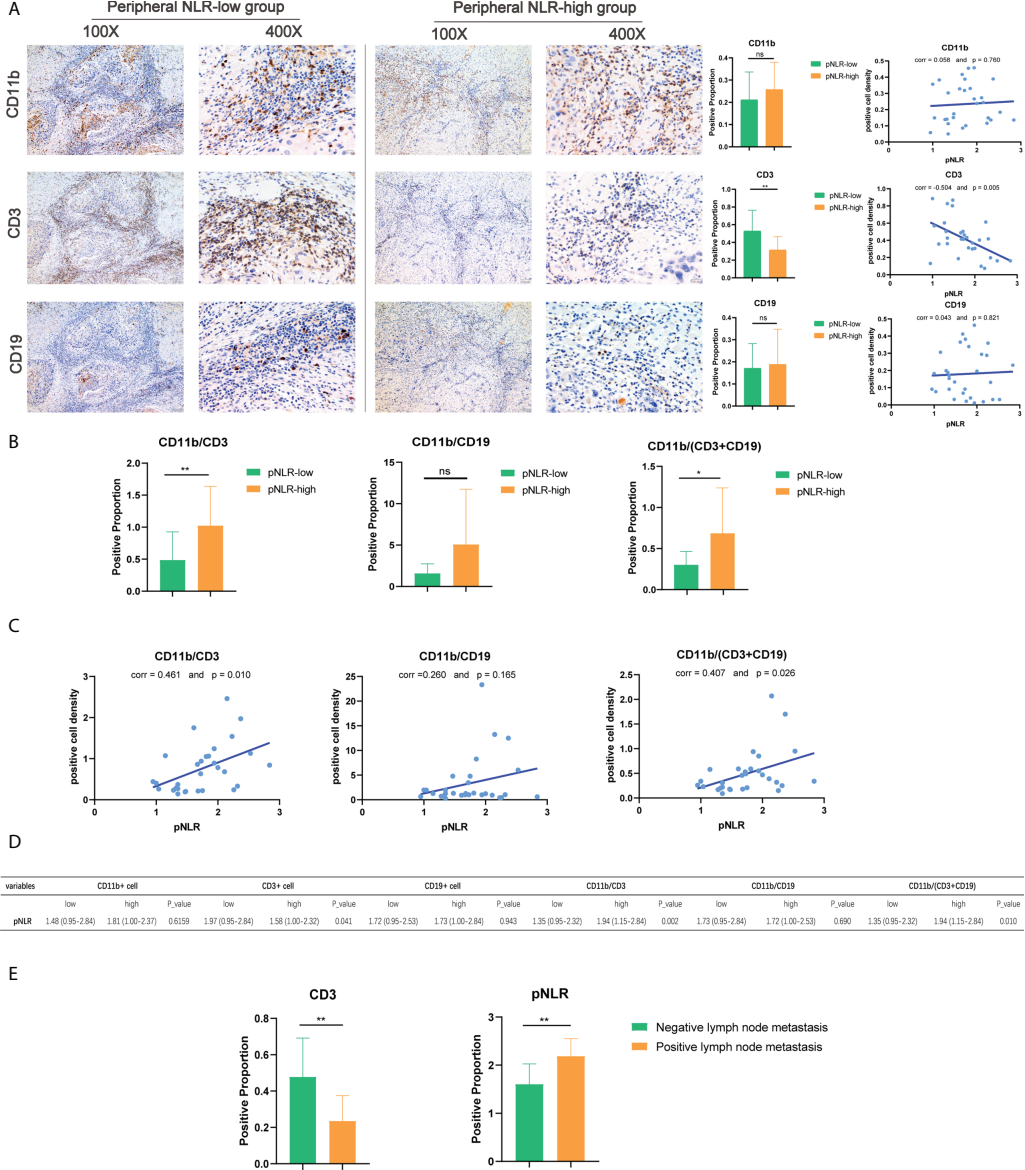
Immunohistochemistry (IHC) of CD11b(+), CD3(+), and CD19(+) cell density in HNSCC tissues. **(A)** Representative images, the Mann–Whitney U test, and Pearson correlation analysis of CD11b(+), CD3(+), and CD19(+) cell density in high-pNLR and low-pNLR groups, presented in ×100 and ×400 magnifications. **(B)** Mann–Whitney U test of CD11b/CD3, CD11b/CD19, and CD11b/(CD3 + CD19) ratios in the tumor tissue between high- and low-pNLR patients. **(C)** Pearson correlation of tumor-infiltrating CD11b/CD3, CD11b/CD19, and CD11b/(CD3 + CD19) ratios and pNLR. **(D)** Mann–Whitney U test of immune marker expression proportion in the TIME between high-pNLR and low-pNLR patients. **(E)** Mann–Whitney U test of the high-pNLR and low-tumor-infiltrating CD3(+) cell density in the lymph node metastasis group and non-lymph node metastasis group. ns, not significant, *p ≤ 0.05; **p ≤ 0.01.

To explore the correlation between pNLR and tNLR distributions in HNSCC samples and the feasibility to predict tumor prognosis in clinical practice, we performed IHC analysis of paired blood samples and tumor specimens in 30 HNSCC patients (see *Methods*). Representative images of CD3(+), CD11b(+) and CD19(+) cells in HNSCC tissue in pNLR-high and pNLR-low groups were presented in 100× and 400× magnification. Of the separate three markers, the density of tumor-infiltrating CD3(+) cells in pNLR-low group was significantly higher than that in pNLR-high group (Mann Whitney U test, *p* = 0.008), while no significant differences were found between pNLR-high and pNLR-low group of CD11b(+) or CD19(+) cell density (Mann Whitney U test, *p* = 0.330, 0.895, respectively) (**Figure 6A** **)**. The distribution of pNLR was shown in **Figure S3**.

As shown in **Figures 6B–D**, there were significant differences in the composite indexes tumor-infiltrating CD11b/CD3 and CD11b/(CD3+CD19) ratios between pNLR-high and pNLR-low patients (Mann–Whitney U test, *p* = 0.007, 0.011, respectively). Consistent with these findings, the pNLR showed positive correlations with the CD11b/CD3 and CD11b/(CD3+CD19) ratios (Pearson correlation, r = 0.461 and *p* = 0.010; r = 0.407 and *p* =0.026, respectively), which indicated that the pNLR was positively associated with the tNLR (**Figures 6B–D**). Moreover, we also found that the pNLR was positively associated with lymph node metastasis ((Mann–Whitney U test, *p* = 0.004), while the tumor-infiltrating CD3(+) density was negatively associated with lymph node metastasis (Mann–Whitney U test, *p* = 0.004) (**Figure 6E**, **Table S3** **).**

A positive correlation was found between pNLR and tNLR, suggesting that the pIS is resembled to the TIME. Moreover, both the pNLR and the tumor-infiltrating lymphocyte density were confirmed to be associated with the HNSCC prognosis as they correlated with lymph node metastasis.

## Discussion

pNLR is a prognostic signature of great clinical interests. Thus far, studies have revealed that pNLR is associated with the prognosis of solid tumors including HNSCC, but most of these studies are single center with small sample sizes (5, 12, 21, 24, 37). Our multicenter HNSCC clinical data analysis confirmed pNLR as one of the promising pISs and could predict an HNSCC prognosis, with an elevated pNLR being associated with poor survival. Moreover, most available studies focused on the observation of the correlation between pNLR and the tumor prognosis. The mechanisms underlying the association of high NLR and poor outcome of cancer patients were poorly understood (5).

Having confirmed the accuracy of pNLR, we next explored the potential reasons for pNLR acting as the prognostic signature in HNSCC. Studies have found the correlation of the TIME and tumor prognoses (15, 19, 38). Under a steady state, few immune cells can be found in normal tissue (39), while in a tumor condition, immune cells can be recruited from peripheral blood to the tumor tissue (14, 40, 41). Therefore, we speculated that an elevated pNLR was associated with the alteration of the TIME.

TIME is an intricate network consisting of immune cell composition, cell functions, cytokines, and chemokines (10, 42). During carcinogenesis, the balance of immune homeostasis is disrupted, which accounts for the number and function aberration of immunocytes (43). To explore the relationship between the TIME and an HNSCC prognosis, we collected HNSCC bulk tumor RNA-seq data from public databases.

Advances in high-throughput technology and the CIBERSORT deconvolution algorithm allowed us to get a large amount of HNSCC TIIC data (8, 32). Among TIICs, a high tNLR was found to be significantly associated with poor survival, which was consistent with our peripheral findings (44). Further, GO and KEGG functional enrichment analyses provided some molecular mechanism clues. Results showed that not only the proportion of immune cells was different between good and poor prognosis groups but also their immune-related functions and activated pathways. Consistent with the trend of TIICs, lymphocyte-related genes were enriched in the good-prognosis group and myeloid leukocyte–related genes were enriched in the poor-prognosis group. Good-prognosis patients may have an anti-TIME (15), while patients in the poor- prognosis group may suffer from tumor-induced immunosuppression during tumor genesis and progression (45). Based on the above findings, tNLR was proven to be correlated with the HNSCC prognosis in both cellular and molecular aspects in the TIME (42). An immune-related model was also constructed for HNSCC clinical outcome prediction. The model had a good accuracy in HNSCC prognosis prediction, suggesting that DEIRG expression in tumor tissues had a significant impact on HNSCC survival and indicating that our model could be widely applied in clinic (6, 46–48).

Apart from immune signatures, there are some clinical factors acting as prognostic biomarkers in HNSCC such as human papillomavirus (HPV) infection. For HNSCC that happens in the oropharynx, HPV infection is common and HPV-positive patients are reported to have a better prognosis than the HPV-negative population (49). Some studies analyzed the pNLR prognostic value in HPV-positive and -negative HNSCC patients, while no differences were found in terms of the NLR in HPV-positive and HPV-negative patients (50, 51). In our clinical cohorts, the tumor sites of oropharynx were very few and the HPV infection status was rarely available, which make it unable to analyze the prognostic value of an HPV infection.

Finally, in order to confirm the relationship between pNLR and tNLR, paired pIS and tumor samples were collected. Studies found the prognostic impact of lymphocytes, neutrophils, or NLR-related TIMEs, respectively, but they did not investigate the interconnections between the pNLR and the tNLR (9, 29, 30). Results confirmed a strong correlation between the pNLR and the tNLR, indicating a resemblance of the pNLR and the TIME (7, 52). Due to the limited sample size, lymph node metastasis was chosen as an objective indicator, which was closely related to the prognosis. Results showed that both pNLR and TIICs had a significant association with lymph node metastasis. We will continue to collect samples and expand the sample size in the follow-up study to verify this conclusion.

A reliable prognostic signature is essential in individual risk quantification and stratification, which are of great concern to clinicians. The pNLR is an easily measurable objective parameter that can be widely used for effective prognostic prediction (25, 53). This study sheds light on the possible mechanisms underlying the predictive value of the pNLR in HNSCC from a unique perspective, that pIS resembles the TIME, by determining the correlation and prognostic value of the pNLR and the TIME. Our results provided a basis for pIS use in clinical practice. There were also some limitations on our study. Due to the small sample size of our clinical cohort, the CD19+ B-cell and CD11b+ myeloid-cell densities showed no statistical difference between groups. Further studies on the biological mechanism underlying the pNLR are also needed.

## Conclusion

An elevated pNLR is associated with poor survival and is positively associated with the tNLR in HNSCC. The pNLR is a feasible prognostic signature that reflects the TIME patterns to some extent in HNSCC.

## Data availability statement

The original contributions presented in the study are included in the article/**Supplementary Material**.

## Ethics statement

The studies involving human participants were reviewed and approved by the Ethics Committee of Hospital of Stomatology, SYSU. The patients/participants provided their written informed consent to participate in this study. Written informed consent was obtained from the individual(s) for the publication of any potentially identifiable images or data included in this article.

## Author contributions

ZH, JZ, and TW were involved in the design and conception. ZH, JZ, HL, and BJ conducted the acquisition of data, statistical analysis, and interpretation of data. ZH, JZ, YPL, and TW drafted the paper. YZL, ZX, and BC retrieved the relevant literatures and revised the paper. All authors contributed to the article and approved the submitted version.

## Funding

This work was supported by the key project of National Natural Science Foundation of China (No. 81630025) and the Science and Technology Program of Guangzhou, China (No. 202206080009).

## Acknowledgments

We thank the patients and investigators who participated in our clinical cancer centers and TCGA and GEO for providing data.

## Conflict of interest

The authors declare that the research was conducted in the absence of any commercial or financial relationships that could be construed as a potential conflict of interest.

## Publisher’s note

All claims expressed in this article are solely those of the authors and do not necessarily represent those of their affiliated organizations, or those of the publisher, the editors and the reviewers. Any product that may be evaluated in this article, or claim that may be made by its manufacturer, is not guaranteed or endorsed by the publisher.

## Supplementary material

Supplementary Figure 1 Distribution plot of pNLR in the clinical cohort. The distribution plot of pNLR in the multicenter clinical cohort (n = 599).

Supplementary Figure 2 Prognostic analysis of the reduced model. ROC and Kaplan–Meier OS curves of the reduced model (b, including age and pathology N stage) on OS at 1–5-year follow-up.

Supplementary Figure 3 Distribution plot of pNLR in clinical IHC cohort. The distribution plot of pNLR in the clinical cohort for further IHC validation (n = 30).

## Abbreviations

HNSCC, head and neck squamous cell carcinoma; pIS, peripheral immune signature; TIME, tumor immune microenvironment; pNLR, peripheral neutrophil-to-lymphocyte ratio; pLMR, peripheral lymphocyte-to-monocyte ratio; TIICs, tumor-infiltrating immune cells; tNLR, tumor-infiltrating neutrophil-to-lymphocyte ratio; AJCC, American Joint Committee on Cancer; TN stage, tumor and lymph node stage; OS, overall survival; ROC, receiver operating characteristic; AUC, area under the curve; TCGA, The Cancer Genome Atlas; NCBI, National Center for Biotechnology Information; GEO, Gene Expression Omnibus; RNA-seq data, RNA sequence data; RPKM, reads per kilobase million mapped reads; DEGs, differentially expressed genes; IRGs, immune-related genes; DEIRGs, differentially expressed immune-related genes; GO-BP, biological process; CC, cellular component; MF, molecular function; FFPE, formalin-fixed paraffin-embedded; IHC, immunohistochemical; HPV, human papillomavirus.
