# Supplementary figures and images for: Peripheral immune signature resembles tumor microenvironment and predicts clinical outcomes in head and neck squamous cell carcinoma
Source: Front Immunol. 2022 Sep 6;13:915207. doi: 10.3389/fimmu.2022.915207 (PMC9486472; doi:10.3389/fimmu.2022.915207)

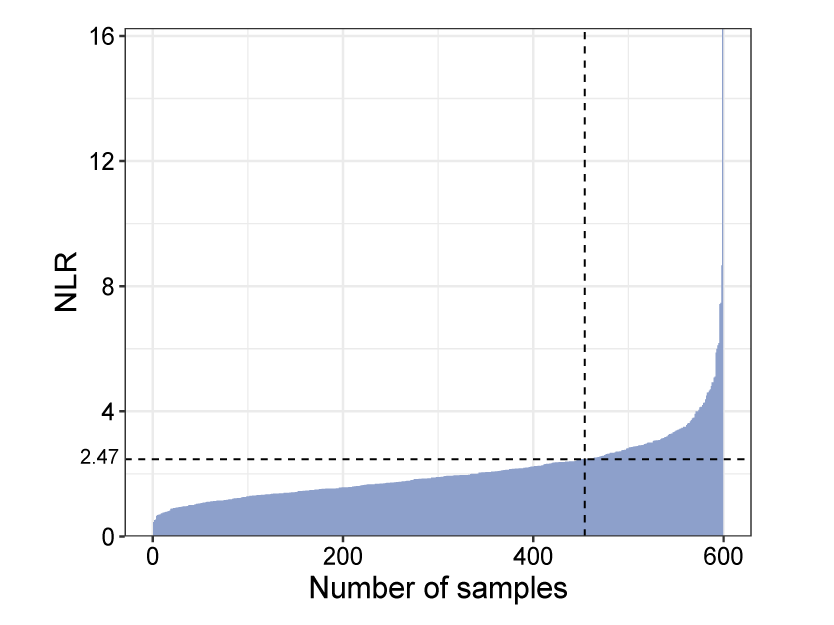

Supplement: Supplementary Figure 1 — Distribution plot of pNLR in the clinical cohort. The distribution plot of pNLR in the multicenter clinical cohort (n = 599). [file Image_1.tif]

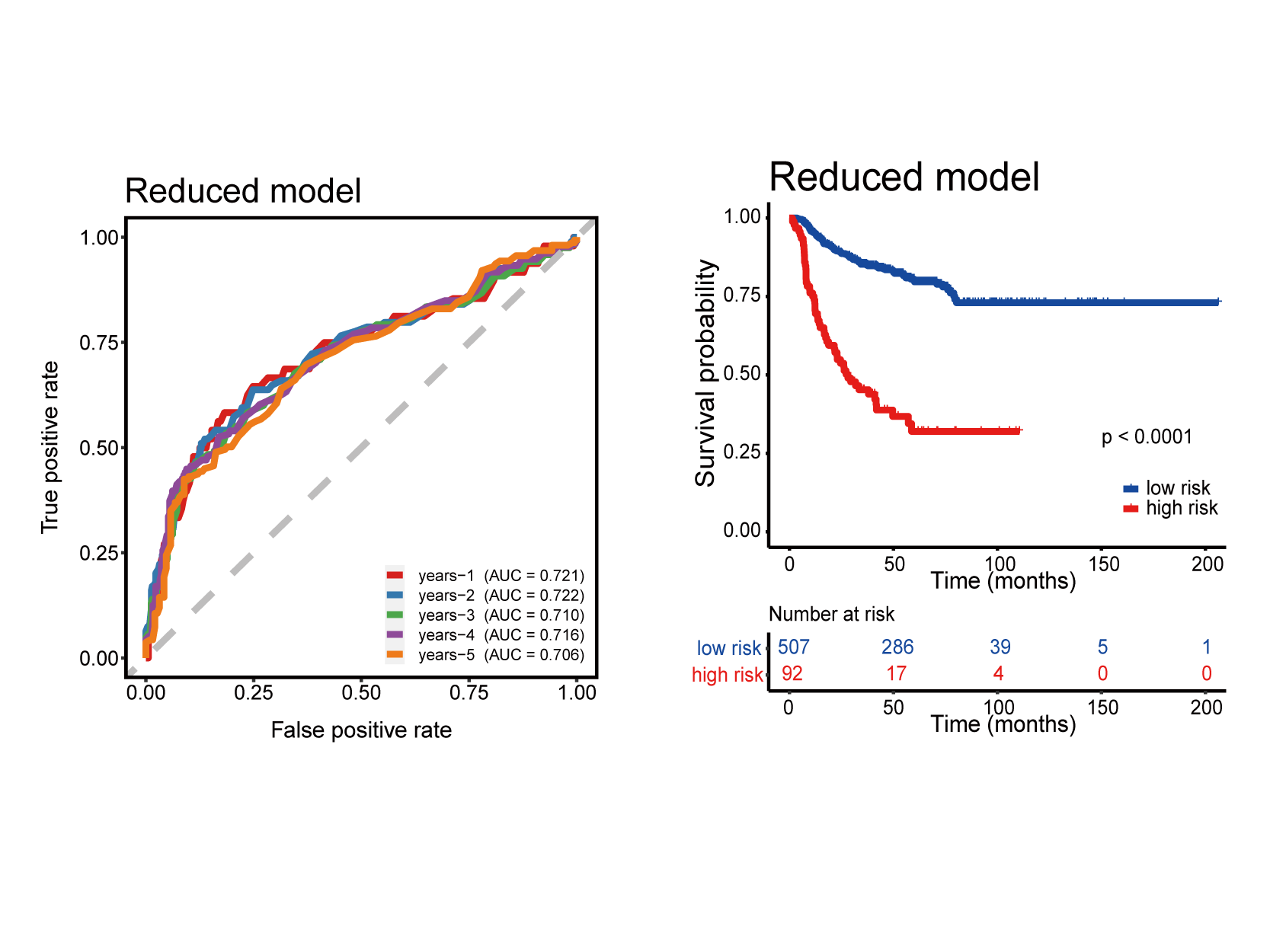

Supplement: Supplementary Figure 2 — Prognostic analysis of the reduced model. ROC and Kaplan–Meier OS curves of the reduced model (b, including age and pathology N stage) on OS at 1–5-year follow-up. [file Image_2.tif]

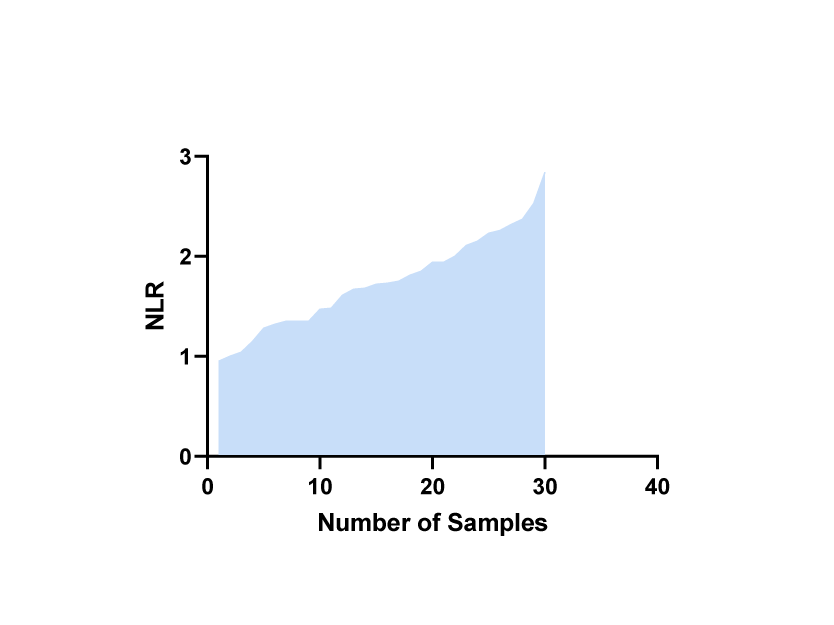

Supplement: Supplementary Figure 3 — Distribution plot of pNLR in clinical IHC cohort. The distribution plot of pNLR in the clinical cohort for further IHC validation (n = 30). [file Image_3.tif]
